# Mass balance, metabolic disposition, and pharmacokinetics of a single oral dose of regorafenib in healthy human subjects

**DOI:** 10.1007/s00280-017-3480-9

**Published:** 2017-11-29

**Authors:** Michael Gerisch, Frank-Thorsten Hafner, Dieter Lang, Martin Radtke, Konstanze Diefenbach, Adriaan Cleton, John Lettieri

**Affiliations:** 1 Bayer AG, Wuppertal, Germany; 2 Bayer AG, Berlin, Germany; 3 Bayer HealthCare Pharmaceuticals, Whippany, NJ USA

**Keywords:** Regorafenib, Metabolism, Biotransformation, Human, Phase I

## Abstract

**Purpose:**

To evaluate the mass balance, metabolic disposition, and pharmacokinetics of a single dose of regorafenib in healthy volunteers. In addition, in vitro metabolism of regorafenib in human hepatocytes was investigated.

**Methods:**

Four healthy male subjects received one 120 mg oral dose of regorafenib containing approximately 100 µCi (3.7 MBq) [^14^C]regorafenib. Plasma concentrations of parent drug were derived from HPLC–MS/MS analysis and total radioactivity from liquid scintillation counting (LSC). Radiocarbon analyses used HPLC with fraction collection followed by LSC for all urine samples, plasma, and fecal homogenate extracts. For the in vitro study, [^14^C]regorafenib was incubated with human hepatocytes and analyzed using HPLC–LSC and HPLC–HRMS/MS.

**Results:**

Regorafenib was the major component in plasma, while metabolite M-2 (pyridine *N*-oxide) was the most prominent metabolite. Metabolites M-5 (demethylated pyridine *N*-oxide) and M-7 (*N*-glucuronide) were identified as minor plasma components. The mean concentration of total radioactivity in plasma/whole blood appeared to plateau at 1–4 h and again at 6–24 h post-dose. In total, 90.5% of administered radioactivity was recovered in the excreta within a collection interval of 12 days, most of which (71.2%) was eliminated in feces, while excretion via urine accounted for 19.3%. Regorafenib (47.2%) was the most prominent component in feces and was not excreted into urine. Excreted metabolites resulted from oxidative metabolism and glucuronidation.

**Conclusions:**

Regorafenib was eliminated predominantly in feces as well as by hepatic biotransformation. The multiple biotransformation pathways of regorafenib decrease the risk of pharmacokinetic drug–drug interactions.

**Electronic supplementary material:**

The online version of this article (10.1007/s00280-017-3480-9) contains supplementary material, which is available to authorized users.

## Introduction

Regorafenib is an orally active multikinase inhibitor that blocks the activity of a broad range of protein kinases involved in tumor angiogenesis, oncogenesis, metastasis, and tumor immunity [1–3]. Antitumor activity with an acceptable safety profile was observed in early clinical studies in patients with a range of solid tumors [4–8]. Subsequent phase III studies demonstrated clinical benefit in patients with refractory metastatic colorectal cancer, advanced gastrointestinal stromal tumors, and hepatocellular carcinoma [9–12], resulting in the approval of regorafenib (160 mg daily for the first 3 weeks of a 4-week cycle) for the treatment of these cancers.

Regorafenib, 4-[4-({[4-chloro-3-(trifluoromethyl)phenyl]carbamoyl}amino)-3-fluorophenoxy]-*N*-methylpyridine-2-carboxamide, is a 1,3-diphenylurea analog composed of a pyridine moiety via one ether linker [1]. After a single dose, metabolites M-2 and M-5 had a signficantly lower exposure than regorafenib, but exhibited similar exposure as regorafenib after multiple doses; therefore, both M-2 and M-5 are regarded as major plasma metabolites [7, 8]. The plasma concentration–time profiles of regorafenib, M-2, and M-5 at steady state exhibited multiple maxima, with an initial maximum (*t*_max_) at 1–6 h, and secondary and tertiary maxima at about 6–8 and 24 h, respectively. The terminal half-lives (*t*_1/2_) of regorafenib and M-2 ranged from 20 to 40 h, whereas M-5 had a longer *t*_1/2_ of about 60 h. Based on the present data, two major metabolites (M-2 and M-5) as well as several minor metabolites have been identified so far in plasma [1]. In addition, M-2 and M-5 show similar in vitro kinase inhibition profiles and comparable potency to regorafenib [13].

The aim of this study was to evaluate the metabolic disposition, mass balance, excretion, and pharmacokinetics of a single oral dose [120 mg, 100 µCi (3.7 MBq)] of [^14^C]regorafenib in healthy male subjects. The regorafenib dose, 120 mg administered as solution, was selected to account for the higher exposure reached with a solution formulation relative to the recommended 160 mg daily dose with the tablet formulation [7, 8]. In addition, we report the metabolism of regorafenib performed in vitro in human hepatocytes.

## Materials and methods

### Labeled regorafenib and reference compounds

Carbon-14-labeled regorafenib, [^14^C]BAY 73-4506, 4-[4-({[4-chloro-3-(trifluoromethyl)phenyl]carbamoyl}amino)-3-fluorophenoxy]-*N*-methylpyridine-2-[^14^C]carboxamide, at a specific activity of 2.53 MBq/mg and radiochemical purity of 99.4% as assessed by high-performance liquid chromatography (HPLC) with radioactivity detection, stable isotope-labeled regorafenib, [^2^H_3_^15^N]BAY 73-4506, 4-[4-({[4-chloro-3-(trifluoromethyl)phenyl]carbamoyl} amino)-3-fluorophenoxy]-*N*-[^2^H_3_]methylpyridine-2-carbox-[^15^N]amide, and [^14^C]-labeled metabolite M-2 were synthesized by the isotope chemistry group at the Department of Drug Metabolism and Pharmacokinetics, Bayer AG (Wuppertal, Germany). In this study, [^14^C]-labeled metabolite M-7 was isolated from urine. Authentic, non-radiolabeled regorafenib was supplied by Bayer AG, Global Chemical and Pharmaceutical Development (Wuppertal, Germany). Authentic standards of known metabolites of regorafenib (M-2, M-3, M-4, M-5, and M-6) were synthesized at Bayer AG, Global Chemical and Pharmaceutical Development. Metabolites M-7 and M-8 were isolated from the current study (Online Resource Appendix A). Ultima Gold™ high flash point scintillation cocktail (6013329) and Ultima Flo™ AP high flash point scintillation cocktail (6013599) were purchased from Packard Instrument BV Chemical Operations (Groningen, The Netherlands). Human hepatocytes were obtained from KaLy-Cell (Illkirch, France) and Hepacult GmbH (Unterföhring, Germany). All other chemicals and solvents used were of reagent grade or better and were obtained from commercial suppliers.

### In vitro metabolism

Primary human hepatocytes were obtained from two male donors. The fresh hepatocytes had a viability of > 80% and were incubated at a density of 1 million cells/mL with [^14^C]regorafenib (2 µM) in William’s E medium at 37 °C in suspension culture. Regorafenib was added from a 200 µM stock solution in acetonitrile, resulting in 1% final acetonitrile in the incubation. At 0 and 2 h, aliquots of the incubation mixture were removed and the incubation was terminated with acetonitrile to a final content of about 30% (v/v). Samples were analyzed directly or stored at − 20 °C until analysis. Analysis was performed by HPLC–liquid scintillation counting (HPLC–LSC) and HPLC–high-resolution mass spectrometry (HPLC–HRMS/MS).

### Subjects and dosing

The clinical part of this study was a 14-day, open-label, phase I study conducted at Charles River Clinical Services Ltd (Edinburgh, UK; EudraCT number: 2010-019120-31). The study was conducted between March 2, 2011, and December 19, 2011. Male subjects aged between 30 and 65 years with a body mass index between 18 and 30 kg/m^2^, deemed healthy on the basis of medical history and a pre-study physical examination, electrocardiogram, and clinical laboratory tests, were included. Informed consent was obtained from all individual participants included in the study. All procedures performed in studies involving human participants were in accordance with the ethical standards of the institutional and/or national research committee and with the 1964 Helsinki Declaration and its later amendments or comparable ethical standards.

Subjects were screened within the 28 days before scheduled drug administration and entered the clinical unit 1 day before drug administration. After an overnight fasting, subjects received a single oral 120 mg dose of regorafenib, containing approximately 100 μCi (3.7 MBq) [^14^C]regorafenib, as a 6 mL solution in an aqueous PEG-based vehicle (Diluent Sol 30 mL oral, Bayer HealthCare AG) followed by 240 mL water. The associated radiation exposure fell within International Commission on Radiological Protection Guidelines for Category IIa studies (0.1–1 mSv) [14].

Discharge from the unit was scheduled after the final examination at 240 h post-dose, contingent on total excretion of more than 95% of administered radioactivity and/or excretion of less than 1% of administered radioactivity in the previous 24-h collection interval. The latest time of discharge was set to 336 h post-dose.

### Safety assessments

Blood pressure, pulse rate, electrocardiogram, physical examination, urine drug screening, and adverse events were evaluated throughout the study. The investigator obtained and recorded all the observed or reported adverse events, the severity (mild, moderate, or severe) of the events, and the relationship to study drug (in the investigator’s opinion).

### Sample collection and preparation

Blood and plasma: Blood samples (18.7 mL per each time point) were collected into nonbeaded lithium-heparinized tubes immediately pre-dose and at the following post-dose times: 0.5, 1, 1.5, 2, 2.5, 3, 4, 6, 9, 12, 16, 20, 24, 36, 48, 72, 96, 120, 144, 192, 240, and 288 h. For each sample, 1 mL of whole blood was stored at 4 °C for subsequent LSC and the remainder was processed for plasma. Individual 150 μL plasma samples for each time point were treated with formic acid (20 μL) and acetonitrile (450 μL). The samples were homogenized using a MS2 Minishaker^®^ (IKA^®^ Labortechnik, Staufen im Breisgau, Germany). The precipitated proteins were removed by centrifugation (5 min, 13,000 rpm, Biofuge). After concentrating the supernatant to about 80 μL, methanol (15 μL) was added. The individual samples were analyzed by HPLC with off-line radioactivity detection for metabolite profiling.

Urine: Urine samples were collected at 12-h intervals post-dose on Day 1 and then at 24-h intervals until 288 h post-dose. Urine was stored at 4 °C until it was divided into aliquots for LSC and subsequent metabolite profiling (aliquots were stored at − 20 °C).

Feces: Fecal samples were collected and homogenized in water on a daily basis until 12 days post-dose. Duplicate aliquots of the homogenates were taken for combustion with oxygen (in preparation for LSC), and separate aliquots were used for metabolite profiling.

### Determination of total radioactivity

Liquid samples: Duplicate aliquots of liquid samples were diluted with 1 mL distilled water, if necessary, and mixed with 10 mL Aquasafe 500 Plus scintillation fluid. Levels of radioactivity were measured by LSC (PerkinElmer 2100) with automatic quench correction by an external standard channel ratio method at 13 °C using Ultima Gold™ as the liquid scintillation cocktail. Lower limits of detection (LLOD) were 30 μg-Eq/L for plasma (sample volume 0.5 mL), 50 μg-Eq/L for whole blood (sample volume 0.3 mL), and 0.016 μg-Eq/g for urine (sample weight 1 g).

Fecal samples: Duplicate aliquots of fecal homogenate (about 0.3 g) were weighed into Combustocones^®^ containing Combustopads^®^ for combustion analysis. Samples were combusted using a Packard Tri-Carb 307 Automatic Sample Oxidizer. The resultant ^14^CO_2_ generated was collected by absorption in Carbo-Sorb (8 mL) to which Permafluor E+ (10 mL) was added. Radioactivity was quantified using a liquid scintillation analyzer (PerkinElmer 2100), with automatic quench correction using an external standard method as urine sample analysis. Samples were allowed to stabilize with respect to heat and light, and each sample was analyzed for 5 min. Prior to calculation of individual results, a background count rate was determined and subtracted from each sample’s count rate. LOD was 0.05 μg-Eq/g (sample weight 0.3 g).

### Determination of regorafenib in plasma

Quantitative analysis of regorafenib in plasma was performed after protein precipitation using a fully validated HPLC–MS/MS assay [15]. The analyses were performed in accordance with the 2001 FDA guidelines on bioanalytical validation [16]. [^2^H_3_,^15^N]-regorafenib was used as internal standard (final concentration: 12.5 µg/L). The calibration range was from 2.00 µg/L to 2.00 mg/L. Quality control samples in the concentration range 5.00 µg/L to 20.0 mg/L were determined with an accuracy of 98.1–103% and a precision of 1.55–10.0% (*n* = 6–8/concentration). All samples were stored at or below − 15 °C and analyzed within 14 days after sample receipt. Stability data indicated that the analytes were stable for this time period.

### Pharmacokinetic analyses

Pharmacokinetic outcome measures were generated by non-compartmental analysis of plasma concentration–time data using WinNonlin v4.1 (Pharsight Corporation) in conjunction with Kincalc v2.50 (Bayer AG). For individual subjects, primary outcome measures were maximum observed concentration (*C*_max_) and area under the concentration–time curve from time zero to infinity (AUC). Since analysis by HPLC–LSC was only assessable up to 144 h, the percentages of the detected metabolites and regorafenib were calculated on the basis of AUC_(0–144)_. The metabolite or regorafenib percentages were calculated by AUC_(0–144)metabolite or regorafenib_ divided by AUC_(0–144)radioactivity_ calculated from data obtained by HPLC–LSC analysis. Secondary outcomes were: time to *C*_max_ (*t*_max_); apparent terminal elimination half-life (*t*_½_); apparent volume of distribution during the terminal phase after extravascular administration (Vz/*F*); and total body clearance of drug calculated after extravascular administration (e.g., apparent oral clearance) (CL/*F*). AUC was estimated by the sum of partial areas: partial areas were calculated using the linear (if *C*_i_ ≤ *C*_i+1_) or the logarithmic (if *C*_i_ > *C*_i+1_) trapezoidal rules. The quality of the half-life calculation was assessed based on graphs showing the concentration–time profile. For reliable calculation of the half-life, at least three data points (concentration/time values) were used. The extrapolated portion of the AUC from the last measured data point *C*(*t*_*n*_) to infinity was calculated using the concentration *C*_last,calc_, which was calculated from the log-linear regression line used for half-life calculation rather than *C*(*t*_*n*_). In addition, appropriate pharmacokinetic parameters were derived from the total radioactivity-versus-time data in blood and plasma.

### Radiochemical HPLC/metabolite profiling

Due to the limited amount of radiocarbon associated with many of the samples, analyses were conducted using HPLC with fraction collection followed by off-line LSC. Samples were analyzed on a 150 × 3 mm Prodigy^®^ ODS-3 100A, 3 µm column (Phenomenex, Aschaffenburg, Germany) protected by 8 × 3 mm Nucleosil^®^ 100-3 C18 HD guard columns (Macherey–Nagel, Düren, Germany). Metabolites were separated with a gradient of aqueous potassium dihydrogen phosphate (1 g/L, pH 2.0, mobile phase A) versus acetonitrile (mobile phase B) at a flow rate of 0.5 mL/min (gradient: 0% B at 0 min, linear to 50% B at 10 min, linear to 60% B at 25 min, linear to 80% B at 26 min, plateau at 80% B to 30 min). The radiochemical profile of [^14^C]regorafenib-derived radioactivity was determined for all urine samples, plasma extracts, and fecal homogenate extracts. Here, HPLC fractionation into 96-well plates was followed by off-line LSC (Wallac 1450 MicroBeta liquid scintillation analyzer) after adding 150 μL of scintillation cocktail to each well (Ultima-Flo™ AP, PerkinElmer; sampling rate: 10 s/well) using the Wallac 1450 Microbeta™ Plusliquid detector. Reconstructed radiochemical profiles were compiled from the resulting data.

### Incubation with human feces

Under anaerobic conditions, a suspension of fresh human feces (about 0.5 g) in degassed water (final volume 5 mL) was treated with a solution of [^14^C]-labelled metabolite M-2 or metabolite M-7 (1 mg/mL acetonitrile; final concentration: 10 μM). After incubation for 24 h at 37 °C, the incubation mixture was terminated by the addition of acetonitrile (2 mL). After centrifugation (5 min, 3000 rpm), the supernatant (25 μL) was directly subjected to HPLC with the respective off-line radioactivity detection. As negative controls, metabolites M-2 and M-7 were incubated in water for 24 h at 37 °C.

### Structural characterization of metabolites

Selected plasma and fecal extracts, urine, and isolated metabolites M-7 and M-8 (see Online Resource, Appendix A) were analyzed on an HP 1200 HPLC system (Hewlett-Packard, Waldbronn, Germany) coupled to a LTQ-Orbitrap hybrid mass spectrometer (Thermo Fisher Scientific GmbH, Bremen, Germany) equipped with heated electrospray ionization (HESI_2) interface. Structures of metabolites in excreta were confirmed by HPLC–HRMS, nuclear magnetic resonance analysis, and by chromatographic/LC–HRMS/MS comparison with authentic reference samples (M-2 to M-6). The metabolite structures are shown in Table 1.

**Table 1:**
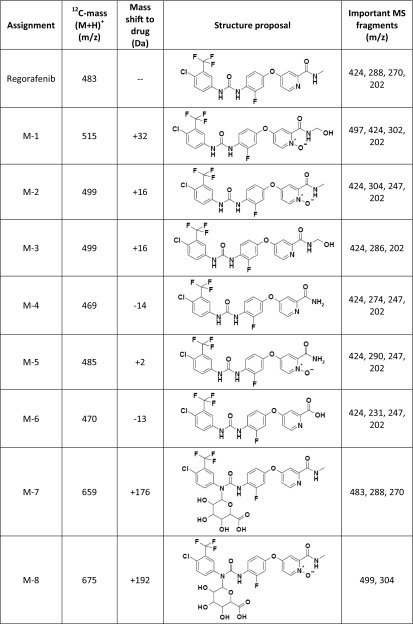
Assignment and proposed structures of the metabolites of regorafenib identified in in vitro incubations and in vivo studies (for further information see Online Resource Appendix C)

## Results

### Demographics, safety, and tolerability

Four healthy male subjects were enrolled in the study, received a single dose of 120 mg regorafenib blended with approximately 100 µCi [^14^C]regorafenib as solution, and completed the study according to protocol. Subjects age ranged from 32 to 46 years [mean 36.8 years (SD 6.3)], body weight ranged from 76 to 92 kg [mean 86.5 kg (SD 7.5)], and body mass index ranged from 24.4 to 27.4 kg/m^2^ [mean 25.8 kg/m^2^ (SD 1.6)]. All were non-smokers (three former smokers) with light or zero alcohol intake. One of the four healthy male subjects reported mild, non-serious headache as a treatment-emergent adverse event that occurred 5 days after administration of the study drug. This event was considered unrelated to the study drug and resolved after treatment with paracetamol. No other adverse events were reported. Electrocardiograms, laboratory investigations, and vital signs showed no clinically relevant changes following regorafenib administration.

### Pharmacokinetics of radioactivity, regorafenib, and metabolites in plasma

**Fig. 1:**
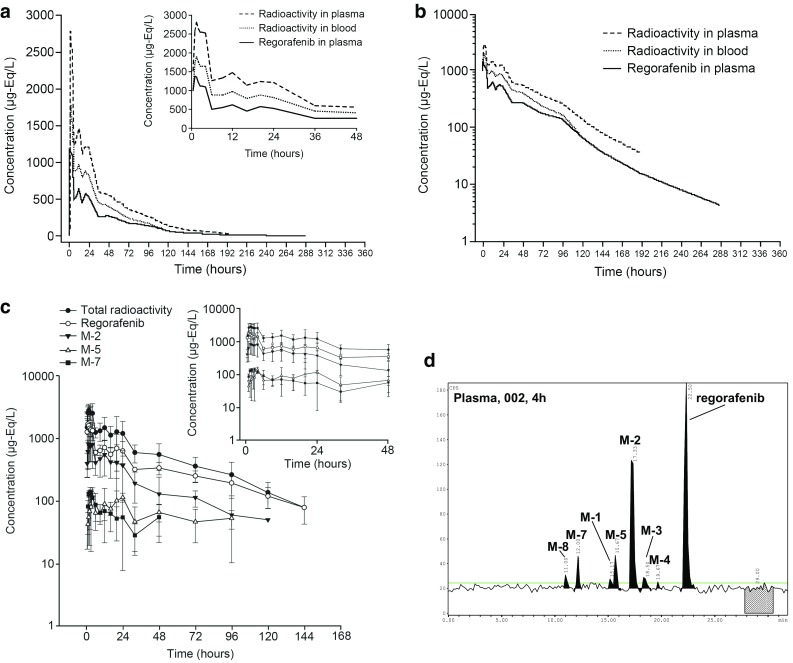
Geometric mean plasma regorafenib concentration–time profile and radioactivity over time following a single oral dose of 120 mg [^14^C]regorafenib solution (*N* = 4). **a** (Linear scale), **b** (logarithmic scale) Regorafenib and radioactivity in plasma and whole blood; **c** regorafenib and metabolites M-2, M-5, and M-7 in plasma; **d** a representative HPLC chromatogram of regorafenib and metabolites in plasma from subject 002

The mean concentration–time profiles in plasma and whole blood are shown in Fig. 1a, b, and the pharmacokinetic parameters of regorafenib in plasma and radioactivity in plasma and whole blood are shown in Table 2. All four subjects showed a similar pharmacokinetic pattern. Following single oral administration, a maximum mean concentration of total radioactivity was observed in both plasma [2.90 mg-Eq/L, %coefficient of variation (%CV) 31] and whole blood (1.95 mg-Eq/L; %CV 19) at 1.5 h post-dose. The concentrations of radioactivity in whole blood generally paralleled with those in plasma, even at a lower level, indicating that regorafenib-derived radioactivity does not bind substantially to red blood cells. The concentration of total radioactivity in both plasma and whole blood appeared to plateau on two occasions, at 1–4 and 6–24 h post-dose (Fig. 1a, b).

**Table 2:** Pharmacokinetic parameters of regorafenib in plasma and total radioactivity in plasma and whole blood after oral administration of a single oral dose of 120 mg [^14^C]regorafenib solution (*N* = 4)

Parameter geometric mean (%CV)	Total radioactivity		Regorafenib
	Plasma	Whole blood	
AUC^a^	84.2 (47)	56.0 (39)	39.0 (34.9)
*C*_max_^a^	2.90 (31)	1.95 (19)	1.58 (36.2)
*T*_max_ (h)^b^	1.50 (1.00–4.00)	1.50 (1.00–4.00)	1.25 (0.500–1.50)
*t*_1/2_ (h)^c^	37.6 (11)	30.4 (8)	48.6 (23.6)
Vz/*F* (L)	77.4 (48)	94.0 (34)	216 (16.3)
CL/*F* (L/h)	1.43 (47)	2.14 (39)	3.08 (34.9)

% of AUC_(144–inf)_: total (2.6%), extrapolated portion

Percent of total AUC_(0–144)_ of radioactivity in plasma based on HPLC–LSC: regorafenib (57.4%), M-2 (28.7%), M-5 (6.3%), and M-7 (3.1%)

^a^mg-Eq·h/L for AUC and mg-Eq/L for *C*_max_ for total radioactivity; mg·h/L for AUC and mg/L for *C*_max_ for regorafenib; values obtained by LC–MS/MS

^b^Median (range)

^c^Due to differences in the lowest limit of quantification (LLOQ) of regorafenib using LC–MS/MS and the lowest limit of detection (LOD) of total radioactivity using liquid scintillation counting (LSC), different intervals for determination of half-life (*t*_1/2_) were applied

Based on metabolite profiling using HPLC–LSC, for all subjects and at all time points investigated, over 89% of the radioactivity present in plasma could be assigned to known structures. A representative chromatogram showing the presence of regorafenib and metabolites in plasma at 4 h post-dose can be found in Fig. 1d.

Concentration–time curves of regorafenib and metabolites M-2, M-5, and M-7 in plasma based on analysis by HPLC–LSC are shown in Fig. 1c. Mean total exposure, AUC_(0–144 h)_, for regorafenib was estimated to be 46.89 mg-Eq·h/L (%CV 21; which is well in line with the data obtained by HPLC–MS/MS), 20.16 mg-Eq·h/L (%CV 59) for M-2, 2.00 mg-Eq·h/L for M-5 (%CV 180), and 2.43 mg-Eq·h/L (%CV 37) for M-7. In all subjects, the plasma concentrations of total radioactivity, regorafenib, and M-2 exhibited several secondary maxima between 1 and 24 h post-dose. For regorafenib, *C*_max_ ranged from 1360 to 2750 μg-Eq/L, for M-2 between 370 and 1480 μg-Eq/L, and for M-5 between 28 and 141 μg-Eq/L. Furthermore, M-5 was detectable over a wide time frame in subjects 002–004, but at four early time points only in subject 001. These data are well in line with data obtained in phase I dose-escalation studies of regorafenib [7]. For M-7, which was detectable by radiochromatography over a wide time frame, *C*_max_ showed no noticeable differences between subjects (137–174 μg-Eq/L). Estimated half-lives of total radioactivity revealed no noticeable inter-subject differences and were in the range of 34–42 h. The half-lives for regorafenib were in the range of those for total radioactivity (39–48 h), except for subject 003 who had a calculated *t*_1/2_ of about 62 h for regorafenib. The differences in half-life estimation between plasma regorafenib and blood and plasma radioactivity may be due to differences in detection levels between liquid scintillation analysis and HPLC–MS/MS. Substantial inter-subject variability was observed in the half-life of M-2, which ranged from about 11 to 33 h. Considering the mean AUC_(0–144 h)_, regorafenib represented 57.4% of total radioactivity and the most prominent metabolite, M-2, represented 28.7%. M-5 and M-7 were identified as minor plasma components, representing 6.3 and 3.1% of mean total radioactivity AUC_(0–144 h)_, respectively. The *N*-methyl hydroxylated pyridine *N*-oxide M-1, the hydroxylated metabolite M-3, the desmethyl metabolite M-4, and M-8 (*N*-glucuronide of M-2) were only occasionally found as traces in plasma.

### Urinary and fecal excretion

**Fig. 2:**
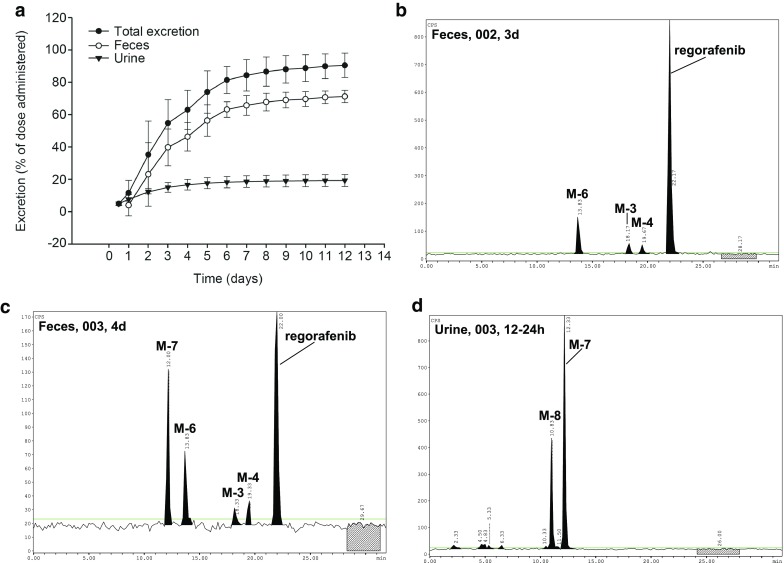
Cumulative excretion of radioactivity (percent of administered dose) following a single oral dose of 120 mg [^14^C]regorafenib solution (*N* = 4). **a** Total radioactivity in feces and urine; **b**–**d** HPLC chromatograms of regorafenib and metabolites in feces and urine from subjects 002 and 003

Following a single oral administration of 120 mg regorafenib, a mean of 90.5% (%CV 3.3, range 86.4–93.0%) of the total administered dose was recovered in combined excreta by the end of the collection period (12 days post dose) (Fig. 2; Table 3). Most of the radioactivity (mean of 71.2% of the administered dose, %CV 5.3, range 68.3–76.3%) was eliminated in feces over the collection period, while excretion via urine over this period accounted for 19.3% of the administered dose (%CV 19, range 16.2–24.7%). Cumulative renal excretion was almost complete by 96 h (mean 16.6%) with less than 3% of the dose renally excreted after this time point. Excretion in feces amounted to 46.3% (mean) within the first 4 days and 67.7% (mean) within 8 days after administration.

**Table 3:** Mean percentages of regorafenib and metabolites excreted in urine and feces following a single oral dose of 120 mg [^14^C]regorafenib solution

Percent of dose (%CV)	Urine	Feces	Total
Excretion (measured radioactivity)	19.3 (19)	71.2 (5.3)	90.5 (3.3)
Excretion by component			
M-8	4.7 (30)	–	4.7 (30)
M-7	12.9 (17)	5.0 (129)	17.9 (32)
M-6	–	14.7 (48)	14.7 (48)
M-3	–	1.8 (36)	1.8 (36)
M-4	–	2.2 (37)	2.2 (37)
Regorafenib	–	47.2 (18)	47.2 (18)
Calculated (sum of components)	17.7	70.8	88.5^a^

^a^Difference from 100% is the result of unknown metabolites and radioactivity not yet excreted

### In vivo metabolite pattern of regorafenib in excreta

The excretion of regorafenib and metabolites in urine and feces is summarized in Table 3. Representative chromatograms showing the presence of regorafenib and metabolites in feces on Day 3 and Day 4 post-dose can be found in Fig. 2b, c, respectively, as well as in urine at 12–24 h post-dose in Fig. 2d. Plasma metabolites M-2 and M-5 were not detected in urine or feces.

Metabolite patterns in urine were similar in all subjects. Renally excreted radioactivity (in total 19.3% of dose) was dominated by two components, the glucuronide metabolites M-7 and M-8, which accounted for a mean of 12.9% (%CV 17) and 4.7% (%CV 30) of the administered dose, respectively. The parent drug could not be detected in urine. Within the first 72 h, several unknown polar metabolites accounted for about 2% of the administered dose. In total, approximately 88–94% of the radioactivity present in 0–288 h urine samples could be assigned to known structures.

In feces (in total 71.2% of dose), the parent drug predominated, accounting for a mean of 47.2% (%CV 18) of the administered dose. M-6 was the second main component in feces with a mean of 14.7% (%CV 48) of the administered dose. Noticeable differences in fecal excretion of M-7 (mean 5.0% of the administered dose, %CV 129) between subjects were observed, ranging from negligible amounts in two subjects (0.03 and 0.3% of the dose) to about 6.0% of the dose and 13.7% of the dose in two other subjects. Additional minor metabolites found in feces were M-3 and M-4, representing about 2% of the administered dose. In total, > 98% of the radioactivity present in feces extracts across all time intervals could be assigned to known structures.

### In vitro metabolite pattern in hepatocytes

The primary biotransformation pathways identified in human hepatocytes were N-oxidation at the pyridine moiety leading to M-2, hydroxylation at the *N*-methyl group leading to M-3, N-demethylation leading to M-4, formation of the carboxylic acid derivative M-6, and glucuronidation of the parent compound leading to M-7 (see Online Resource Appendix B). A combination of these primary metabolic reactions leading to M-1 (N-oxidation and methylhydroxylation), M-5 (N-oxidation and N-demethylation), and M-8 (glucuronidation of M-2) was also observed.

## Discussion

Following a single oral dose of 120 mg regorafenib blended with approximately 100 µCi [^14^C]regorafenib to four healthy volunteers, the pharmacokinetic analysis of total radioactivity consisting of parent drug and circulating metabolites suggests rapid absorption (*t*_max_ ≤ 2 h). The pharmacokinetic parameters reported for regorafenib and metabolites M-2 and M-5 in this study are comparable to previously published data in patients who received a single dose of 120 mg of regorafenib as tablets [7]. The plasma profiles revealed the parent drug to be the major component in terms of mean AUC_(0–144 h)_ (57.4% of total radioactivity), while M-2 was the most prominent metabolite (28.7% of total radioactivity). Metabolites M-5 and M-7 were identified as minor plasma components (6.3 and 3.1% of total AUC_(0–144 h)_ radioactivity). It must be noted that at steady state, mean AUC_*τ*,ss_ and *C*_max_ of metabolites M-2 and M-5 showed similar total systemic exposure compared with the parent drug [7]. Furthermore, the metabolites M-2 and M-5 have demonstrated similar potency to the parent drug in several in vitro and in vivo experiments, suggesting that the metabolites may contribute to the observed clinical activity of regorafenib [1, 13], although it should be noted that estimated free plasma concentrations (fraction unbound in plasma: 0.488% for regorafenib, 0.188% for metabolite M-2, 0.053% for metabolite M-5 [13]) suggest that the parent drug is the main contributor to the clinical activity of regorafenib [17]. To maximize the overall exposure to regorafenib, M-2, and M-5, it is recommended that patients take regorafenib with a low-fat meal [18]. The appearance of several secondary maxima over 24 h of total radioactivity and parent drug, as well as its pyridine *N*-oxide metabolite M-2, gave hints for enterohepatic circulation [19]. The following processes are considered to be responsible for enterohepatic circulation: biotransformation of regorafenib to the primary metabolites pyridine *N*-oxide metabolite M-2 and *N*-glucuronide M-7 in the liver; biliary excretion of parent drug and metabolites into the gut; and reduction of the pyridine *N*-oxide M-2 as well as hydrolytic cleavage of the *N*-glucuronide M-7 by gut flora (e.g., intestinal microorganisms), yielding the parent drug, which is reabsorbed.

**Fig. 3:**
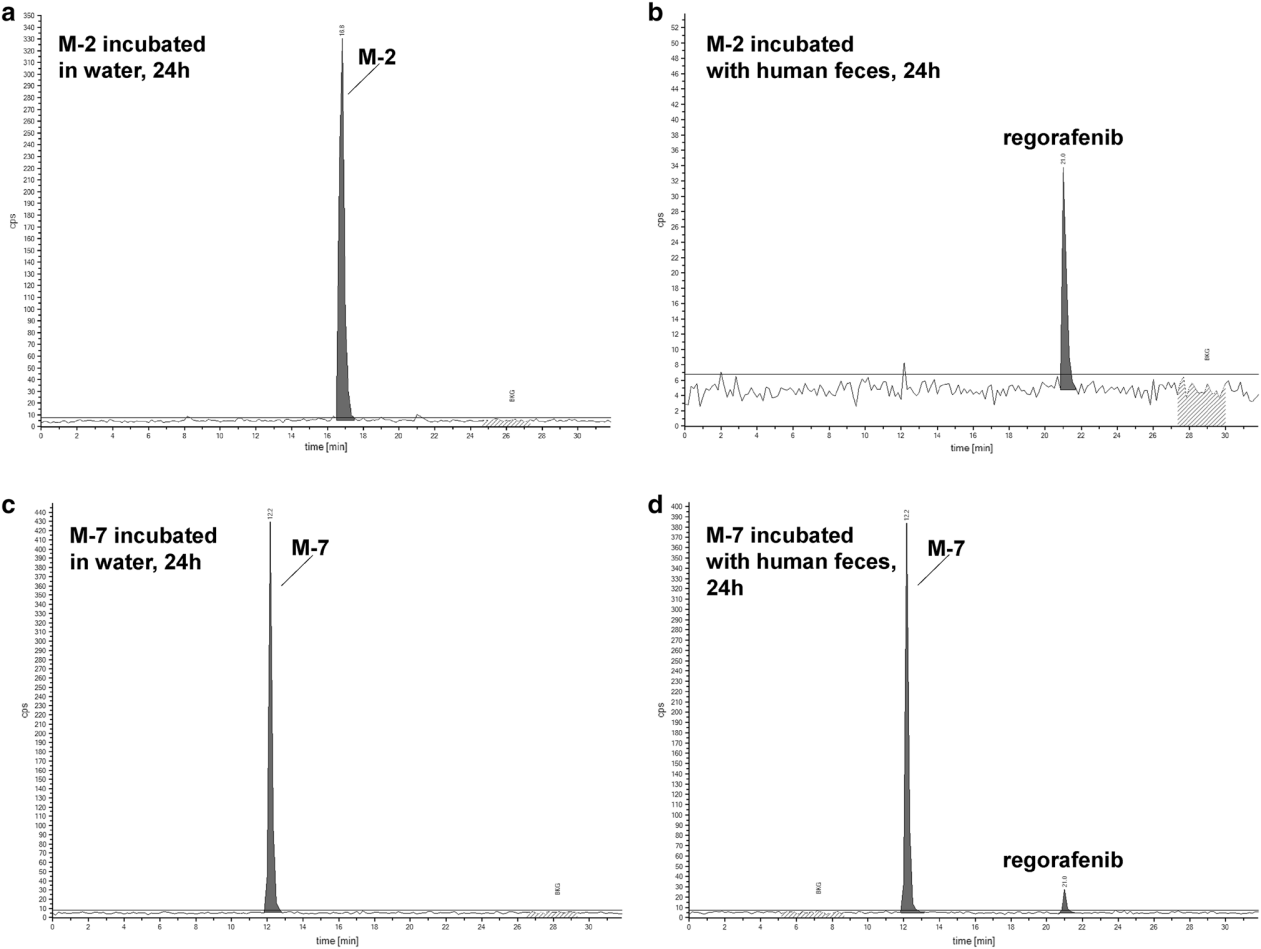
Incubation of metabolites M-2 and M-7 with human feces. **a**–**d** HPLC chromatograms after incubation of metabolites M-2 (**a, b**) and M-7 (**c, d**) with water (**a, c**) and with human feces suspension (**b, d**)

The hypothesis of fecal degradation was supported by several in vitro findings: (a) incubation of the parent drug in human hepatocytes resulted in the formation of M-2 (which is further metabolized to M-8) and M-7 (Online Resource, Appendix B); (b) incubation of M-2 and M-7 in feces under anaerobic conditions (24 h, Fig. 3) resulted in the formation of the parent compound due to reduction of the *N*-oxide or cleavage of the *N*-glucuronide. When M-8 was incubated with human feces, reduction of the *N*-oxide leading to M-7 proceeded much faster than cleavage of the *N*-glucuronide yielding M-2. Consistently, reduction of M-2 to the parent drug occurred faster (quantitative after 24 h incubation in feces) than hydrolysis of M-7 to the parent drug (< 10% under the applied conditions), indicating that M-2 is more sensitive to fecal degradation than M-7. This is supported by the lack of detection of M-2 in the excreted feces of all subjects, compared with detection of M-7 in considerable amounts in the feces of two out of four subjects.

In total, 90.5% of the radioactivity was recovered in the excreta during the collection period (14 days). The amount of radioactivity excreted into urine was 19.3%, whereas most of the radioactivity was excreted via the biliary/fecal route into the feces with 71.2% of the administered dose. Although regorafenib and metabolite M-2 were the major components in plasma, neither compound was detected in urine. However, the corresponding *N*-glucuronides M-7 and M-8 represented almost all of the renally excreted radioactivity (> 90%). Metabolite M-7, which was a minor plasma component, and M-8 were found only occasionally in plasma. Whether effective renal elimination of M-7 and M-8 from plasma, or glucuronidation of the parent drug and M-2 by the kidney tissue, explains these data could not be deduced by this study.

The parent drug was the predominant component in feces, representing about 47% of the administered dose. As aforementioned, pyridine *N*-oxide metabolite M-2, metabolite M-8 (*N*-glucuronide of M-2), and to a lesser degree, *N*-glucuronide M-7, are not stable toward gut flora (intestinal microorganisms). Therefore, radioactivity in feces attributed to the parent drug may actually be derived from the reduction of metabolite M-2, hydrolysis of metabolite M-7, or the parent drug that was either not absorbed or was excreted in bile. Noticeable differences within subjects were observed for the *N*-glucuronide metabolite M-7 in feces, ranging from negligible amounts to about 14%.

**Fig. 4:**
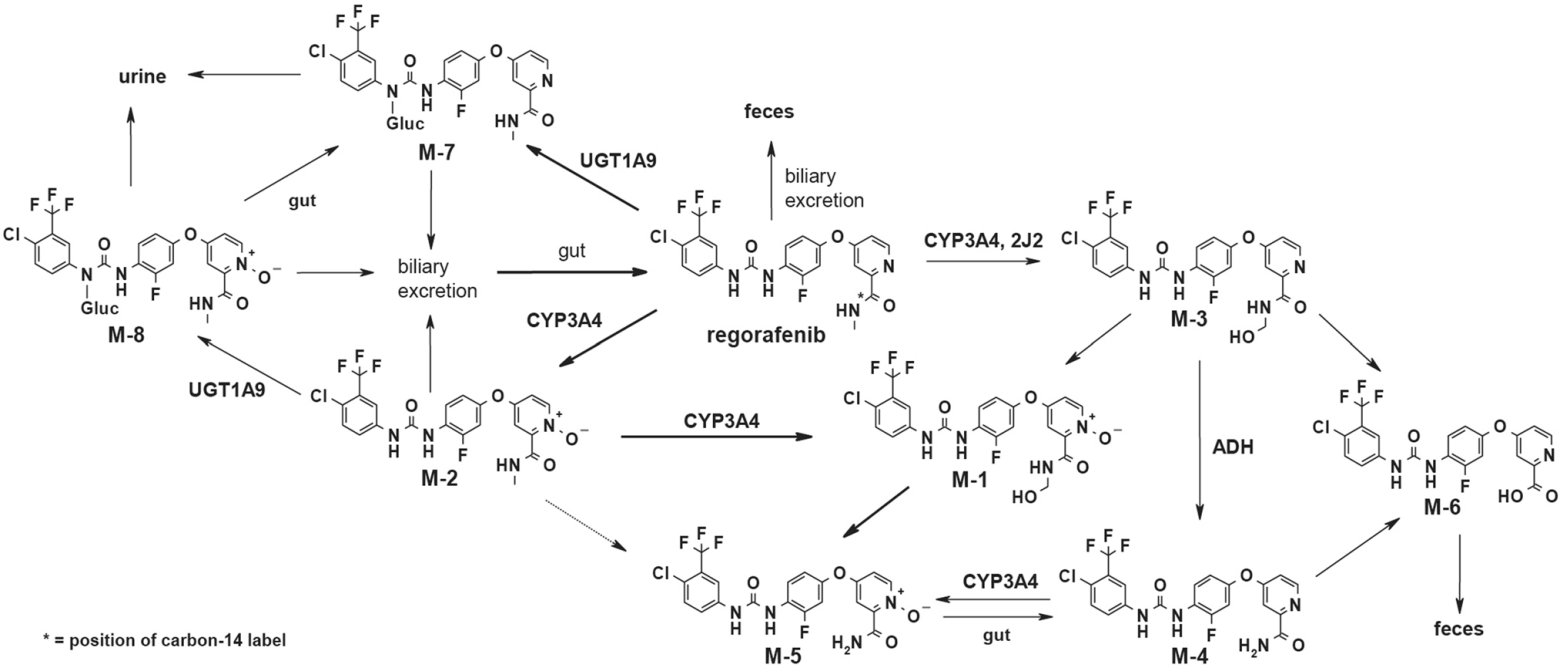
The proposed metabolic pathway of regorafenib in humans in vivo

The carboxylic acid metabolite M-6 was found as the second main component in feces, accounting for about 15% of the administered dose. Formation of the carboxylic acid metabolite M-6 may be derived from hydrolysis of the parent drug or from further biotransformation from primary metabolites. Stability investigations in feces (ex vivo) under anaerobic conditions and at physiological pH revealed the parent drug to be stable. Detailed in vitro investigations revealed M-3 and M-4 to be the most likely precursors of M-6 (Fig. 4). Metabolite M-3, formed by methylhydroxylation, is a substrate of alcohol dehydrogenase, yielding the amide M-4 as the main product and M-6 as a minor product (Bayer AG, data on file). In addition, metabolite M-4 was identified as the most suitable substrate for amide hydrolysis, yielding M-6. All in vitro data suggest that M-6 is the metabolic end point of oxidative and hydrolytic pathways of M-3 and M-4, and is not formed by direct hydrolysis of the parent drug. Therefore, M-6 can be regarded as a secondary/tertiary clearance product of regorafenib.

Based on in vitro data, oxidative biotransformation of regorafenib is predominantly catalyzed by CYP3A4 and conjugation with glucuronic acid by UGT1A9 [1, 13]. Oxidative pathways via CYP3A4 with primary metabolites M-2 and M-3, and M-3, M-4, M-6, and M-8 as excreted components, accounted for about 24% of the dose; glucuronidation via UGT1A9 with M-7 as the excreted component accounted for about 18% of the dose. However, overall it should be stated that the fecal instability of metabolites and the apparent enterohepatic circulation of regorafenib complicated the interpretation of the mass balance data and, consequently, the calculation of the substrate fraction metabolized by the respective enzymes to determine the overall contribution of each biotransformation pathway to the metabolic clearance of regorafenib.

The in vitro data with CYP3A4 as major CYP isoform for the formation of metabolites M-2 and M-5 are in line with the results of a clinical drug–drug interaction study, in which co-administration of ketoconazole, a strong CYP3A4 inhibitor, with a single 160 mg dose of regorafenib resulted in greater than a 90% decrease in mean AUC and *C*_max_ of M-2 and M-5 [1, 20]. The same study also revealed an increase of only about 30–40% in mean AUC and *C*_max_ of regorafenib, indicating that alternative clearance mechanisms (other than CYP3A4 and not affected by ketoconazole) for regorafenib play an important role in humans with glucuronidation of regorafenib by UGT1A9 (to yield *N*-glucuronide M-7) and maybe also direct biliary elimination of unchanged regorafenib. This is in line with the interpretation of data obtained by the human mass balance study.

In summary, this study demonstrated that regorafenib was rapidly absorbed following oral dosing and confirmed that enterohepatic circulation plays a role in regorafenib metabolism. Most of the administered radioactive dose was excreted via the biliary/fecal route mainly as regorafenib and to a lesser extent as metabolites resulting from primary oxidative biotransformation or direct glucuronidation. Excretion via urine is a minor route of excretion.

## Electronic supplementary material

Below is the link to the electronic supplementary material.

Supplementary material 1 (PDF 274 KB)
