# Supplementary material for: Mass balance, metabolic disposition, and pharmacokinetics of a single oral dose of regorafenib in healthy human subjects
Source: Cancer Chemother Pharmacol. 2017 Nov 29;81(1):195–206. doi: 10.1007/s00280-017-3480-9 (PMC5754413; doi:10.1007/s00280-017-3480-9)
Supplement: Supplementary file 1 — Supplementary material 1 (PDF 274 KB) [file 280_2017_3480_MOESM1_ESM.pdf]

Online Resource – Cancer Chemotherapy and Pharmacology

Mass balance, metabolic disposition, and pharmacokinetics of a single oral dose of regorafenib in healthy human subjects

Authors

Michael Gerisch[1], Frank-Thorsten Hafner[1], Dieter Lang[1], Martin Radtke[1], Konstanze Diefenbach[2], Adriaan Cleton[2], John Lettieri[3]

Affiliations

[1]Bayer AG, Wuppertal, Germany

[2]Bayer AG, Berlin, Germany

[3]Bayer Healthcare Pharmaceuticals, Whippany, NJ, USA

Corresponding author

Adriaan Cleton

Clinical PK ONC, Bayer AG, 13353 Berlin, Germany

Tel: +49 304 6819 2867

Email: Adriaan.Cleton@bayer.com

Appendix A: Isolation of Metabolites M-7 and M-8

Pooled urine (20 mL; 5 mL each from subject 001–004) from interval 0–12 h was applied onto solid-phase extraction (SPE) (Oasis HLB, 1 g, 20 mL, Waters, Eschborn, Germany). The cartridge was washed successively with water (10 mL), methanol/water mixtures (10 mL, 3:97, v/v; 10 mL, 1:9, v/v; 10 mL, 2:8, v/v, 10 mL, 3:7, v/v, 10 mL, 1:1, v/v, 10 mL, 9:1, v/v) and acetonitrile (10 mL) resulting in nine fractions. After radioactivity analysis, fractions 1–7 were discarded. Fractions 8 and 9 were pooled, concentrated to about 0.3 mL, and forwarded to preparative HPLC (Phenomenex Prodigy 3 ODS(3) column, 150 × 3 mm, 3 µm, ser. no. 522228-1) using phosphate buffer (2 g/L, pH 2.0) as mobile phase A and acetonitrile as mobile phase B. The chromatography was performed in a total of three runs (0.1 mL of the concentrated aqueous solution, each injected for chromatographic separation) using a gradient elution at a flow rate of 0.5 mL/min, starting with A:B (90:10), gradient to A:B (50:50, 0–10 min), gradient to A:B (45:55, 10–15 min), gradient to A:B (10:90, 15–16 min), and isocratic A:B (10:90, 16–20 min) at a flow rate of 0.5 mL/min and at a detection wavelength of 265 nm using DAD and a column temperature of 45°C. The first preparative run was divided into eight fractions. Runs 2 and 3 were divided into three fractions. Fraction 6 from the first run and fraction 3 from runs 2 and 3 were pooled and concentrated under vacuum to about 1 mL (M-7). Fraction 1 from runs 2 and 3 were pooled and concentrated under vacuum to about 1 mL (M-8). The pooled extracts were applied onto SPE for desalination (Oasis HLB, 0.1 g, 1 mL). The cartridges were washed with water (1 mL) and methanol (1 mL). Finally, about 10 µg of M-7 and M-8 was dissolved in DMSO-d6 and analyzed by NMR spectrometry.

Appendix B: HPLC Chromatogram after incubation of [^{14}C]regorafenib in human hepatocytes (2 µM, 2h)

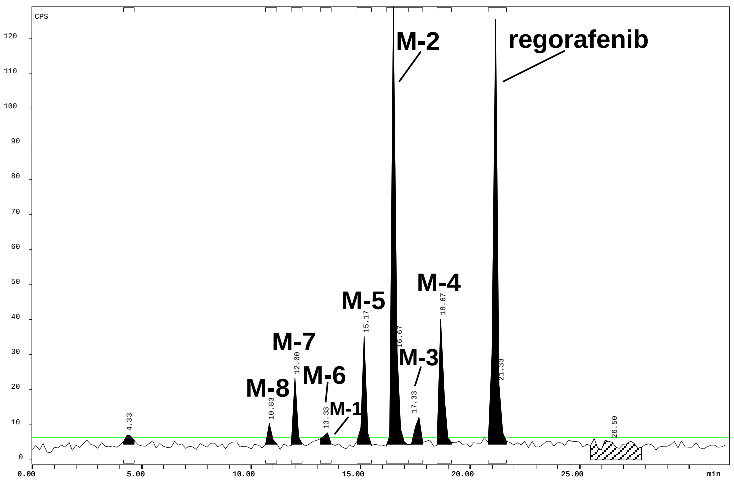

Appendix C: Structural characterization of metabolites

Parent drug (regorafenib)

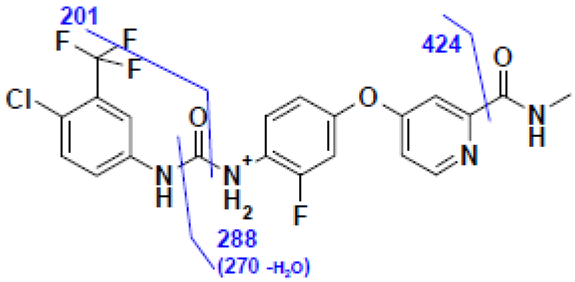

The high-resolution mass spectral data showed [M+H]+ at m/z 483.084. The MS2 product ion spectrum showed major fragment ions at m/z 424.045 (loss of N-methylformamide $CHONHCH_3$) (**Fig. S1**), m/z 288.077, m/z 270.067, and m/z 201.986 with the urea amide bonds as sites of fragmentation.

M-1

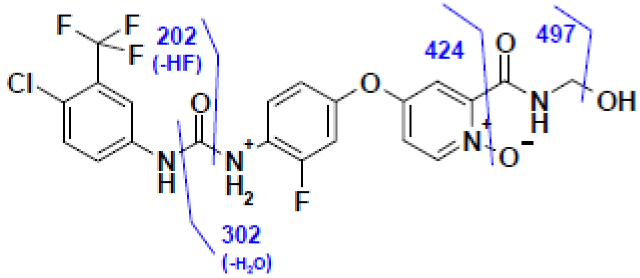

M-1 was found in trace amounts in incubations with human hepatocytes. The high-resolution mass spectral data showed [M+H]+ at m/z 515.073 **(Fig. S2)**, which is 32 Da (+2O) higher the than the parent drug. The MS2 product ion spectrum showed major fragment ions at m/z 497.063 (loss of H_2O), m/z 424.047 (loss of N-hydroxymethylformamide $CHONHCH_2OH$), m/z 302.057, and m/z 201.986 with the urea amide bonds as sites of fragmentation. Therefore, M-1 was interpreted as a pyridine N-oxide and N-methylhydroxylated product (combination of M-2 and M-3) of regorafenib.

M-2 (BAY 75-7495)

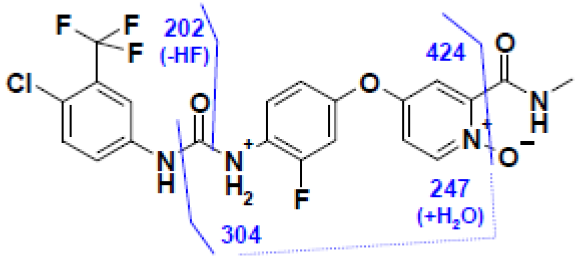

M-2 was found as a major metabolite in incubations with human hepatocytes. The high-resolution mass spectral data showed [M+H]+ at m/z 499.079 (**Fig. S3**), which is 16 Da (+O) higher than the parent drug. The MS2 product ion spectrum showed major fragment ions at m/z 424.045 (loss of N-

methylformamide and oxygen $CHONHCH_3 + O$), m/z 304.072, m/z 247.051, and m/z 201.986 with the urea amide bonds as sites of fragmentation. Therefore, M-2 is interpreted as a pyridine N-oxide product of regorafenib. Furthermore, all mass spectral data were identical to the synthetic reference material BAY 75-7495.

M-3 (BAY 81-8753)

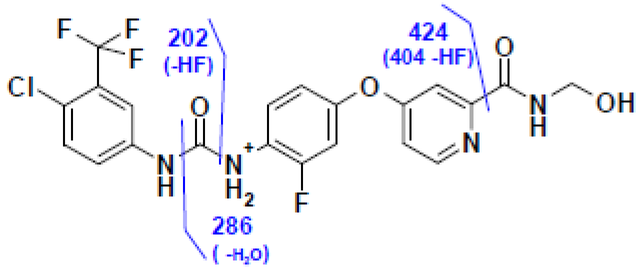

Only small amounts of M-3 were found in human hepatocytes. The high-resolution mass spectral data showed [M+H]+ at m/z 499.077 **(Fig. S4)**, which is 16 Da (+O) higher than the parent drug. The MS2 product ion spectrum showed major fragment ions at m/z 424.045 (loss of N-hydroxymethylformamide $CHONHCH_2OH$), m/z 286.061, and m/z 201.986 with the urea amide bonds as sites of fragmentation. Therefore, M-3 is interpreted as an N-methylhydroxylated product of regorafenib. Furthermore, all mass spectral data were identical to the synthetic reference material BAY 81-8753.

M-4 (BAY 75-1098)

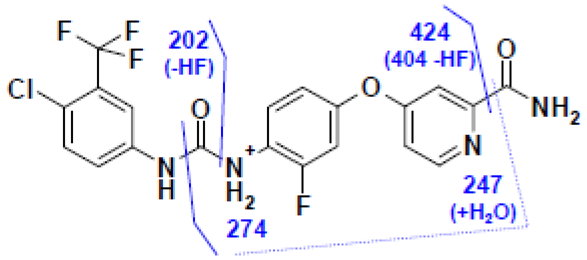

Only small amounts of M-4 were found in human hepatocytes. The high-resolution mass spectral data showed [M+H]+ at m/z 469.067 (**Fig. S5**), which is a loss of 14 Da ($-CH_2$), compared with the parent drug. The MS2 product ion spectrum showed major fragment ions at m/z 424.045 (loss of formamide $CHONH_2$), m/z 274.061, m/z 247.050, and m/z 201.986 with the urea amide bonds as sites of fragmentation. Therefore, M-4 was interpreted as an N-demethylated product of regorafenib. Furthermore, all mass spectral data were identical to the synthetic reference material BAY 75-1098.

M-5 (BAY 81-8752)

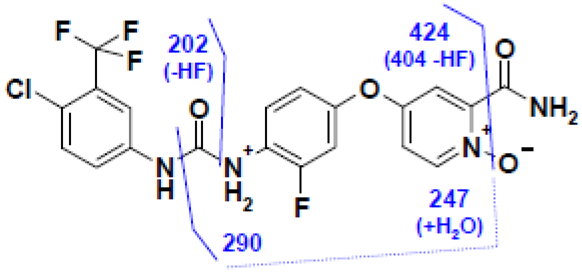

M-5 was found as a metabolite in incubations with human hepatocytes. The high-resolution mass spectral data showed [M+H]+ at m/z 485.061, which is 2 Da (-CH_2, +O) higher than the parent drug. The MS2 product ion spectrum (**Fig. S6**) showed fragment ions at m/z 424.045 (loss of formamide and oxygen $CHONH_2$ and O), m/z 290.056, m/z 247.050, and m/z 201.986 with the urea amide bonds as sites of fragmentation. Therefore, M-5 was interpreted as a pyridine N-oxide and N-demethylated product (combination of M-2 and M-4) of regorafenib. Furthermore, all mass regorafenib 16 spectral data were identical to the synthetic reference material BAY 81-8752.

M-6 (BAY 1005352)

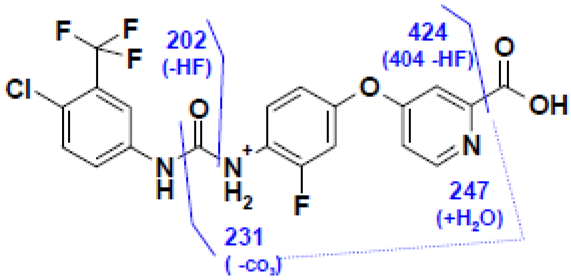

Only small amounts of M-6 were found in human hepatocytes. The high-resolution mass spectral data showed [M+H]+ at m/z 470.051 **(Fig. S7)**, which is a loss of 13 Da (-NH_2CH_3, +H_2O) compared with the parent drug. The MS2 product ion spectrum showed major fragment ions at m/z 424.045 (loss of CO_2), m/z 231.055, m/z 247.050, and m/z 201.986 with the urea amide bonds as sites of fragmentation. Therefore, M-8 was interpreted as an N-demethylated and hydrolyzed carboxylic acid derivative of regorafenib. Furthermore, all mass spectral data were identical to the synthetic reference material BAY 1005352.

M-7

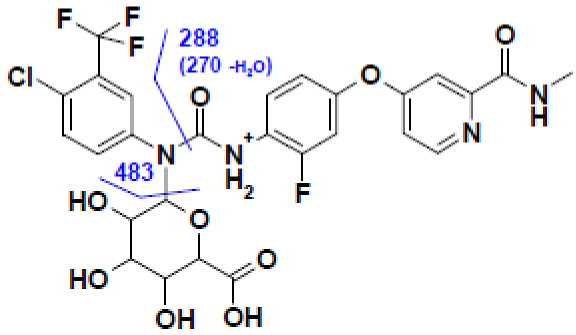

M-7 was found as a metabolite in incubations with human hepatocytes. The high-resolution mass spectral data showed [M+H]+ at m/z 659.113 **(Fig. S8)**, which is 176 Da (+$C_6H_8O_6$) higher than the parent drug. The MS2 product ion spectrum showed fragment ions at m/z 641.103 (loss of H_2O), m/z 483.082 (loss of -$C_6H_8O_6$, anhydro glucuronic acid, 176 Da), which is the pseudomolecular ion of parent drug, m/z 288.077, and m/z 270.067 with the urea amide bonds as sites of fragmentation. Therefore, M-7 was interpreted as a glucuronic acid conjugate of parent drug regorafenib. For further structure elucidation, M-7 was isolated and purified by HPLC from human urine (GCM 2082-01), and subjected to LC-MS and NMR analysis (**Fig. S10**). From these data it was proposed that M-7 is N-glucuronidated at the urea nitrogen adjacent to the trifluoromethyl-chloro phenyl moiety.

M-8

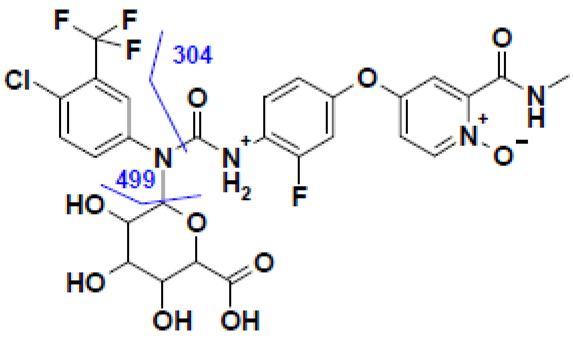

M-8 was found as a metabolite in incubations with human hepatocytes. The high-resolution mass spectral data showed [M+H]+ at m/z 675.109 **(Fig. S9)**, which is 192 Da (+$C_6H_8O_6$, +O) higher than the parent drug. The MS2 product ion spectrum showed fragment ions at m/z 657.099 (loss of H_2O), m/z 499.077 (loss of -$C_6H_8O_6$, anhydro glucuronic acid, 176 Da), which is the pseudomolecular ion of the hydroxylated parent drug, and m/z 304.072 with the urea amide bonds as sites of fragmentation. The fragment ion m/z 304.072 is characteristic for M-2; therefore, M-8 was interpreted as a glucuronic acid conjugate of M-2. For further structure elucidation, M-8 was isolated and purified by HPLC from human urine (GCM 2082-02) and subjected to LC-MS and NMR-analysis. From these data it was proposed that M-8 is N-glucuronidated at the urea nitrogen adjacent to the trifluoromethyl-chloro phenyl moiety.

Fig. S1 Product ion spectrum of regorafenib (BAY 73-4506)

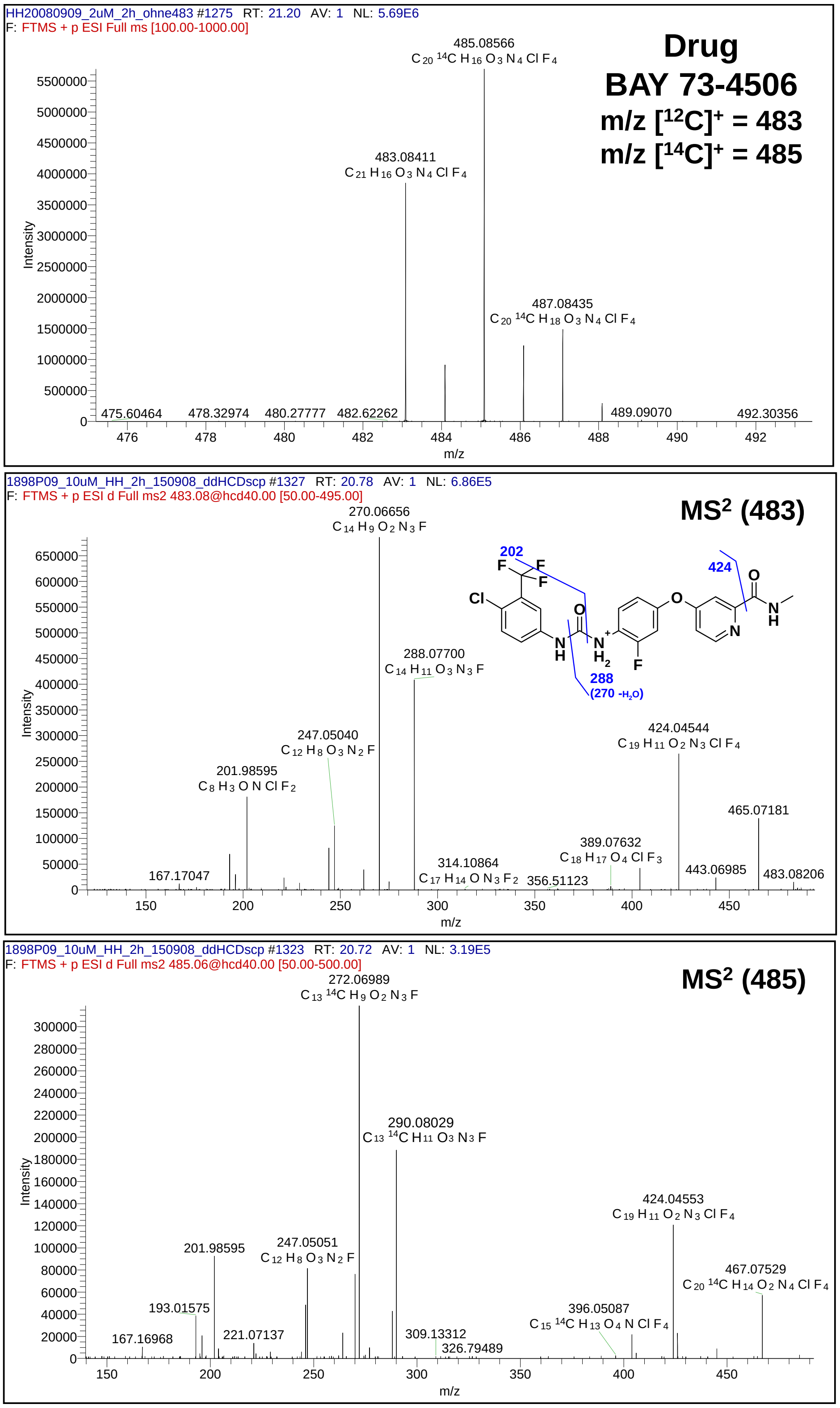

Fig. S2 Product ion spectrum of M-1

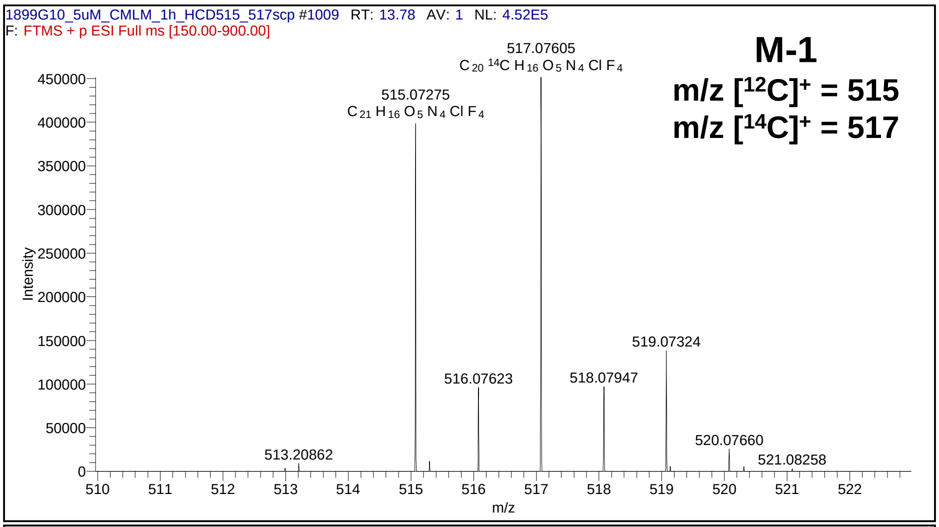

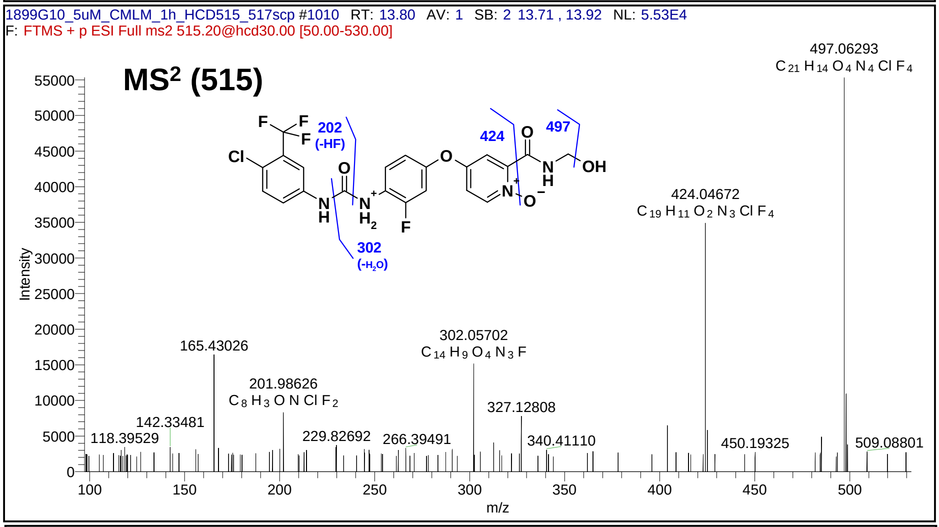

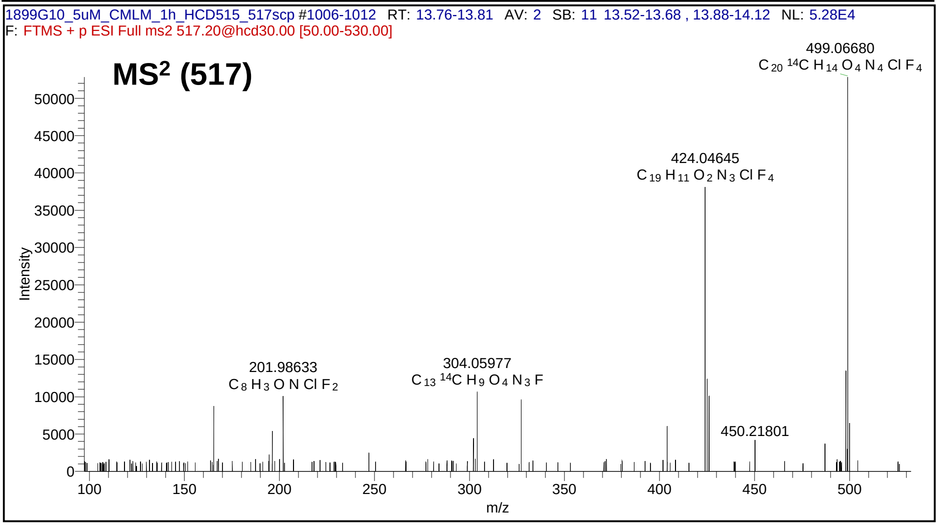

Fig. S3 Product ion spectrum of M-2

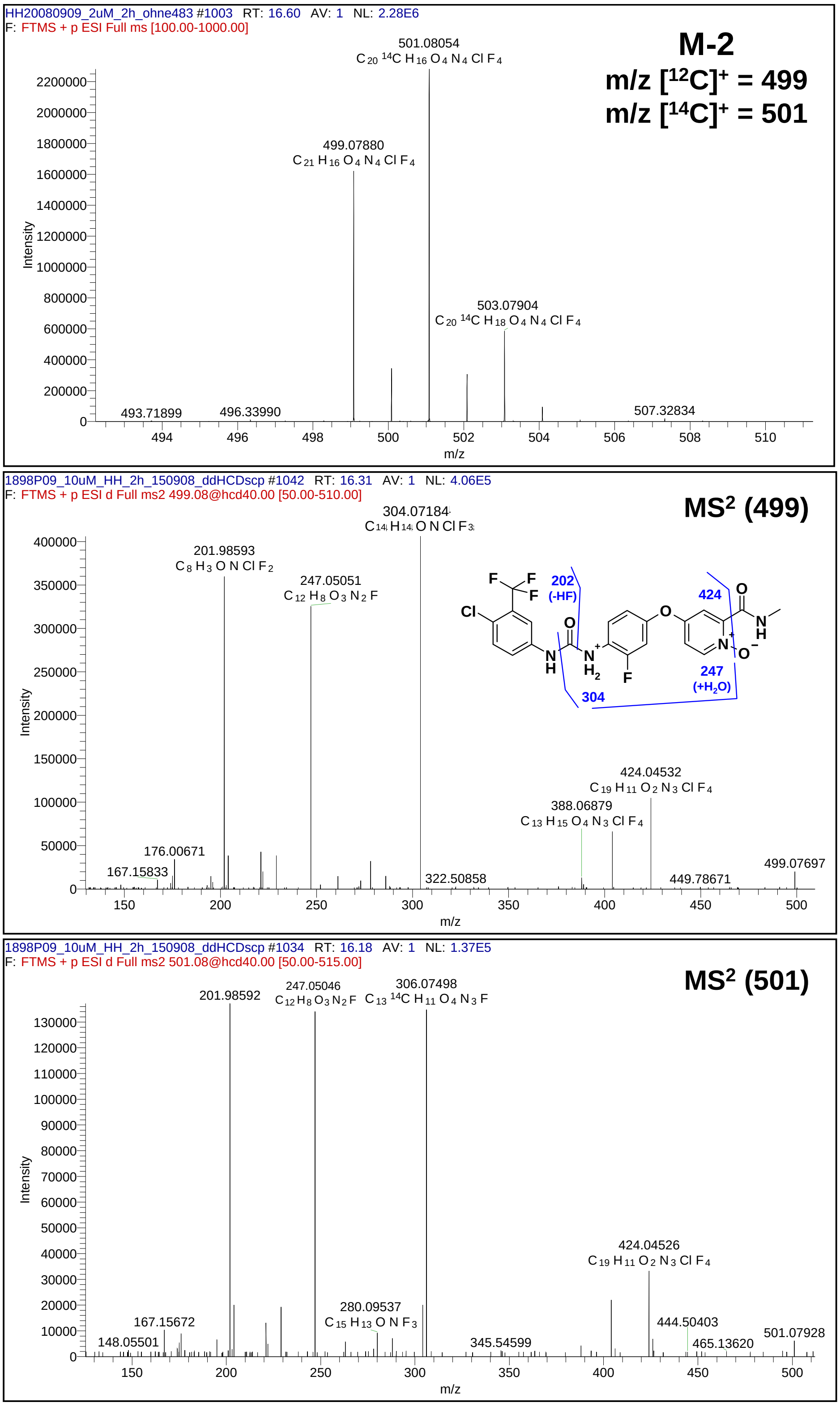

Fig. S4 Product ion spectrum of M-3

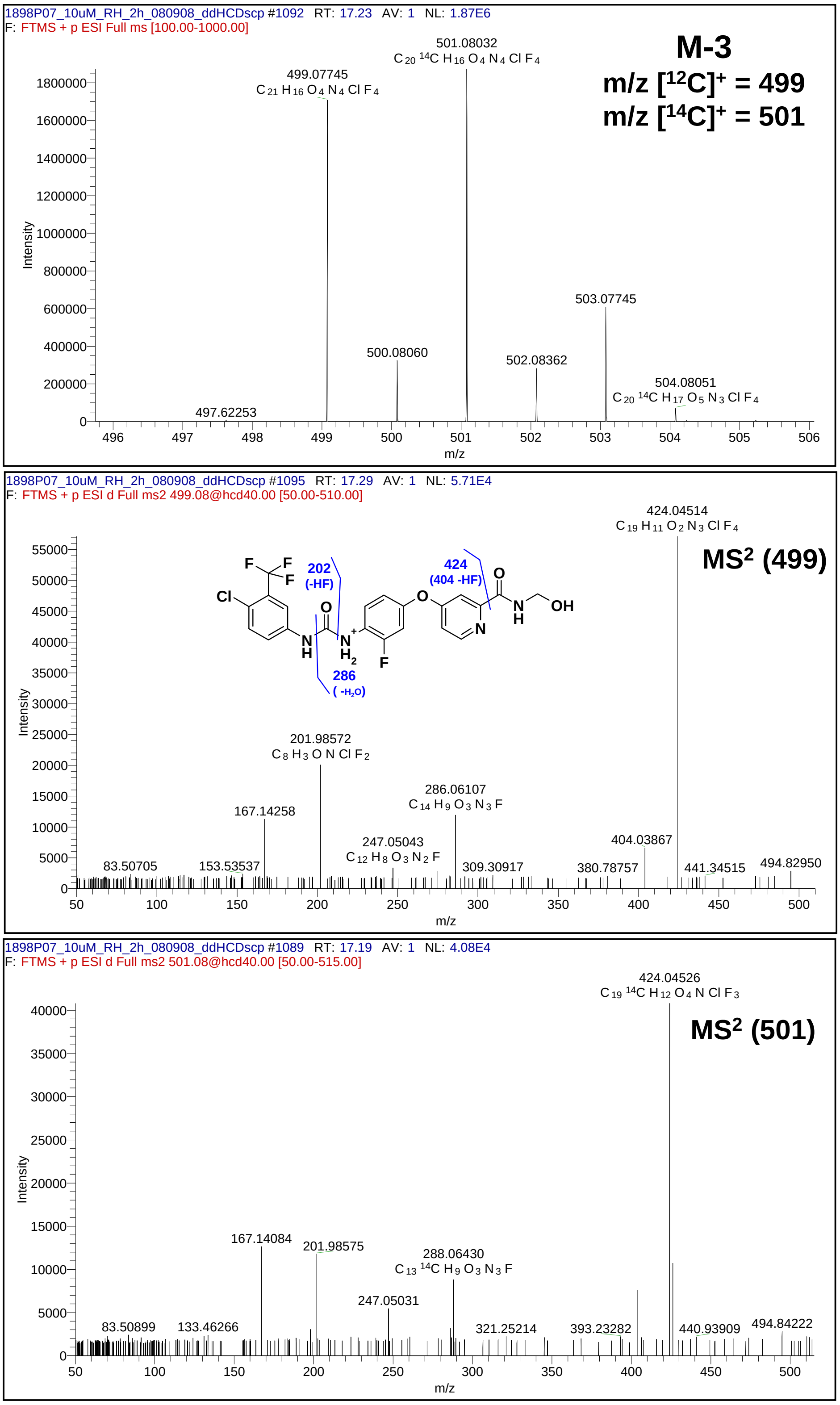

Fig. S5 Product ion spectrum of M-4

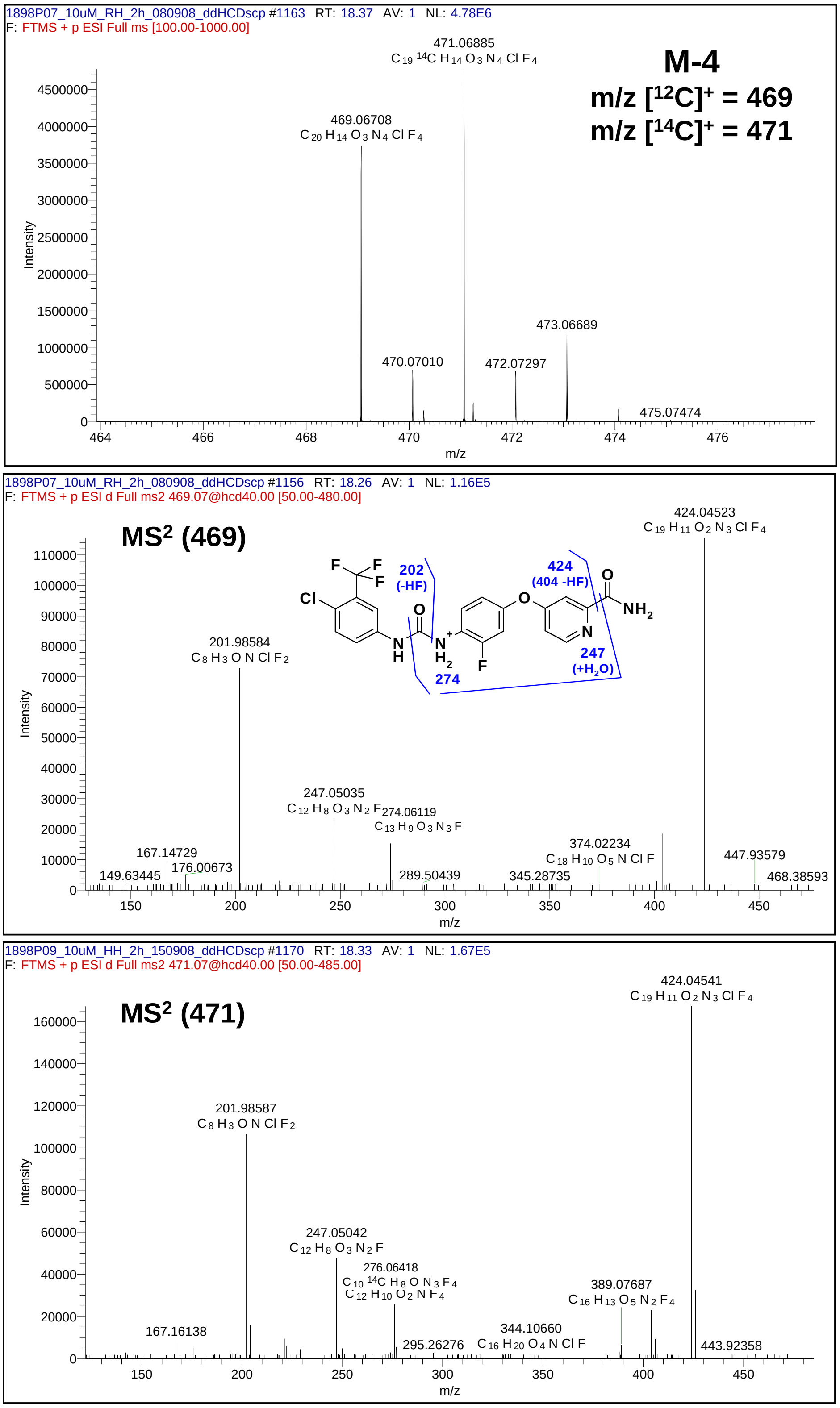

Fig. S6 Product ion spectrum of M-5

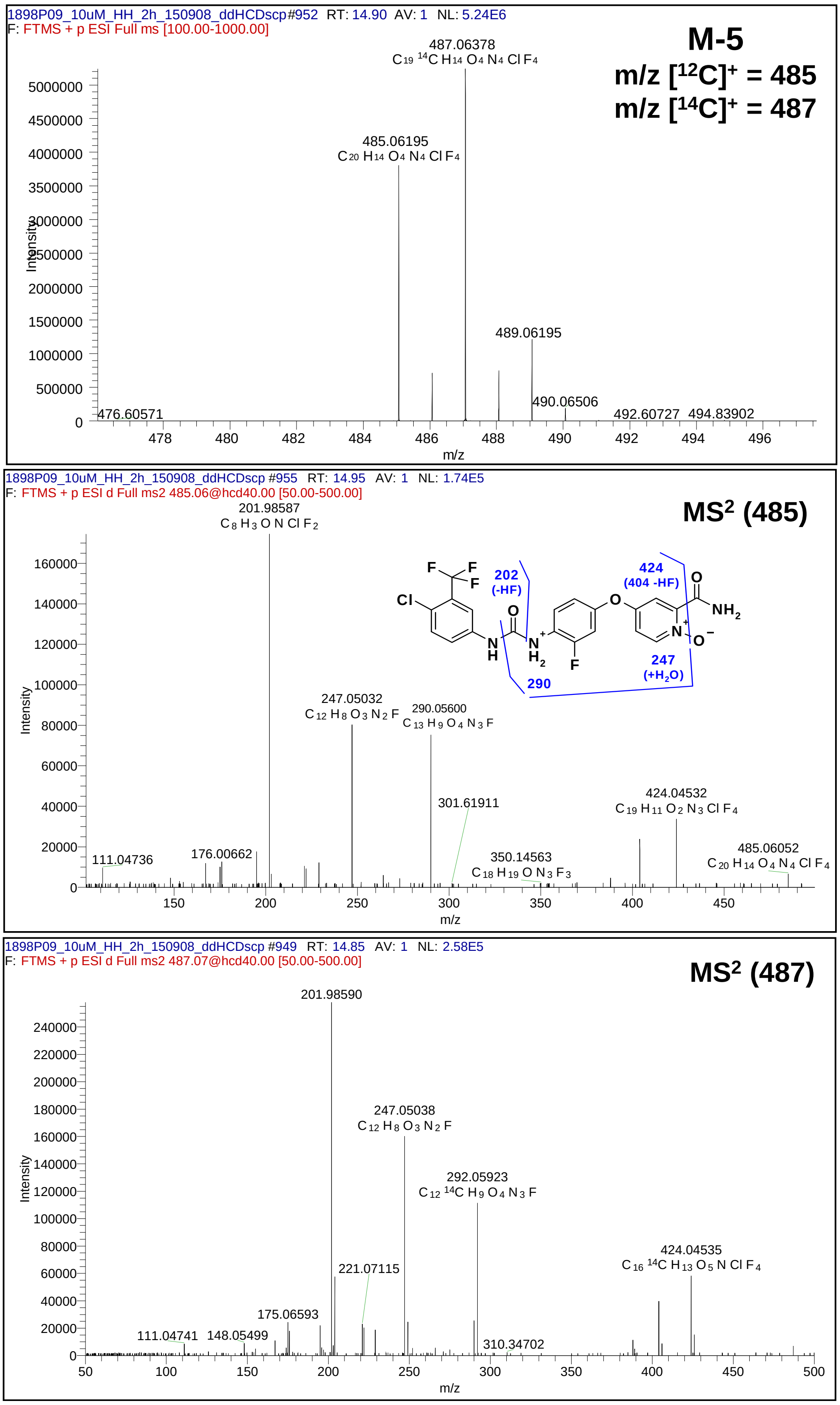

Fig. S7 Product ion spectrum of M-6

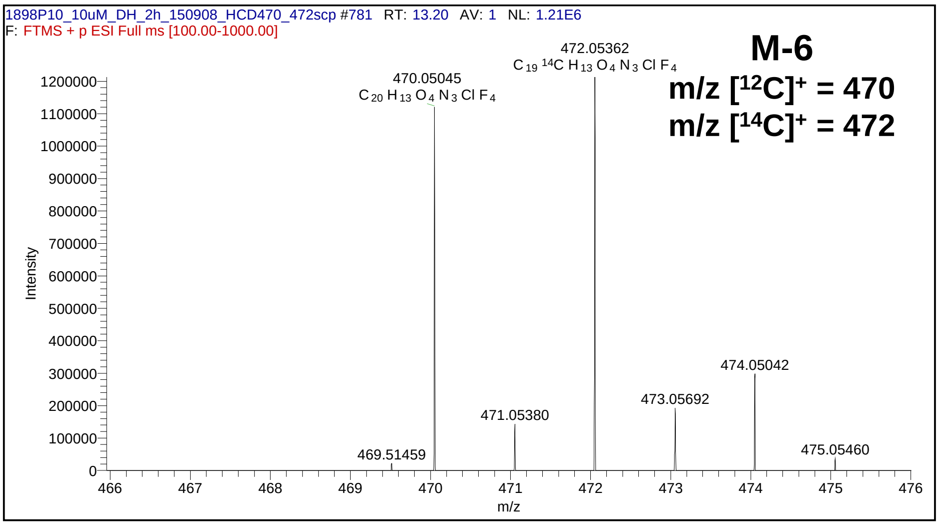

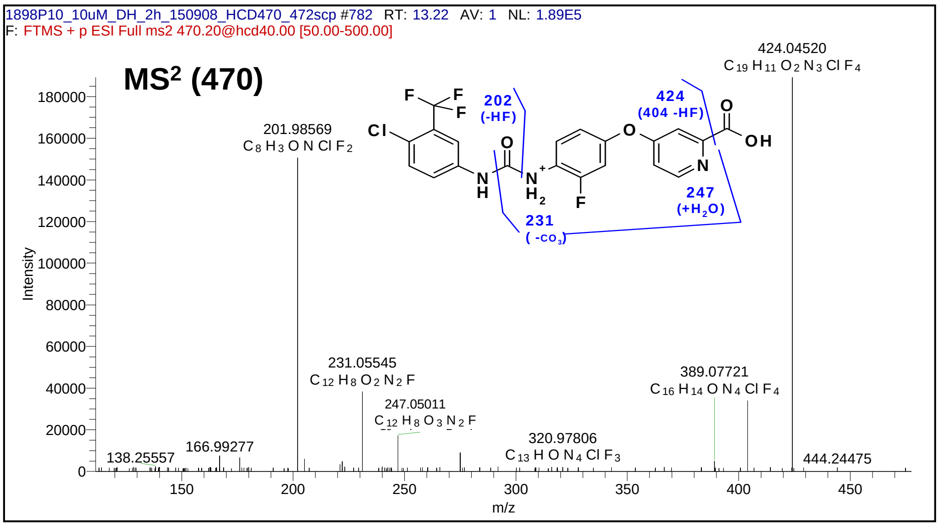

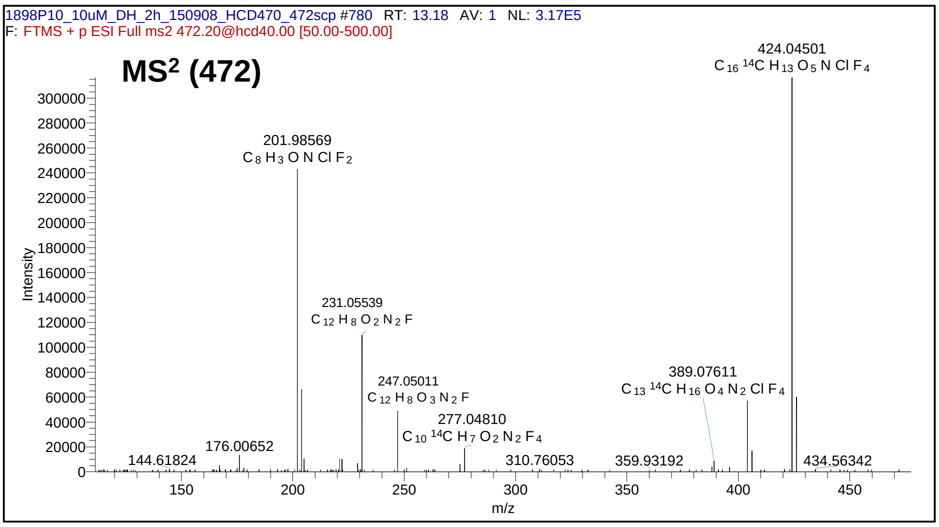

Fig. S8 Product ion spectrum of M-7

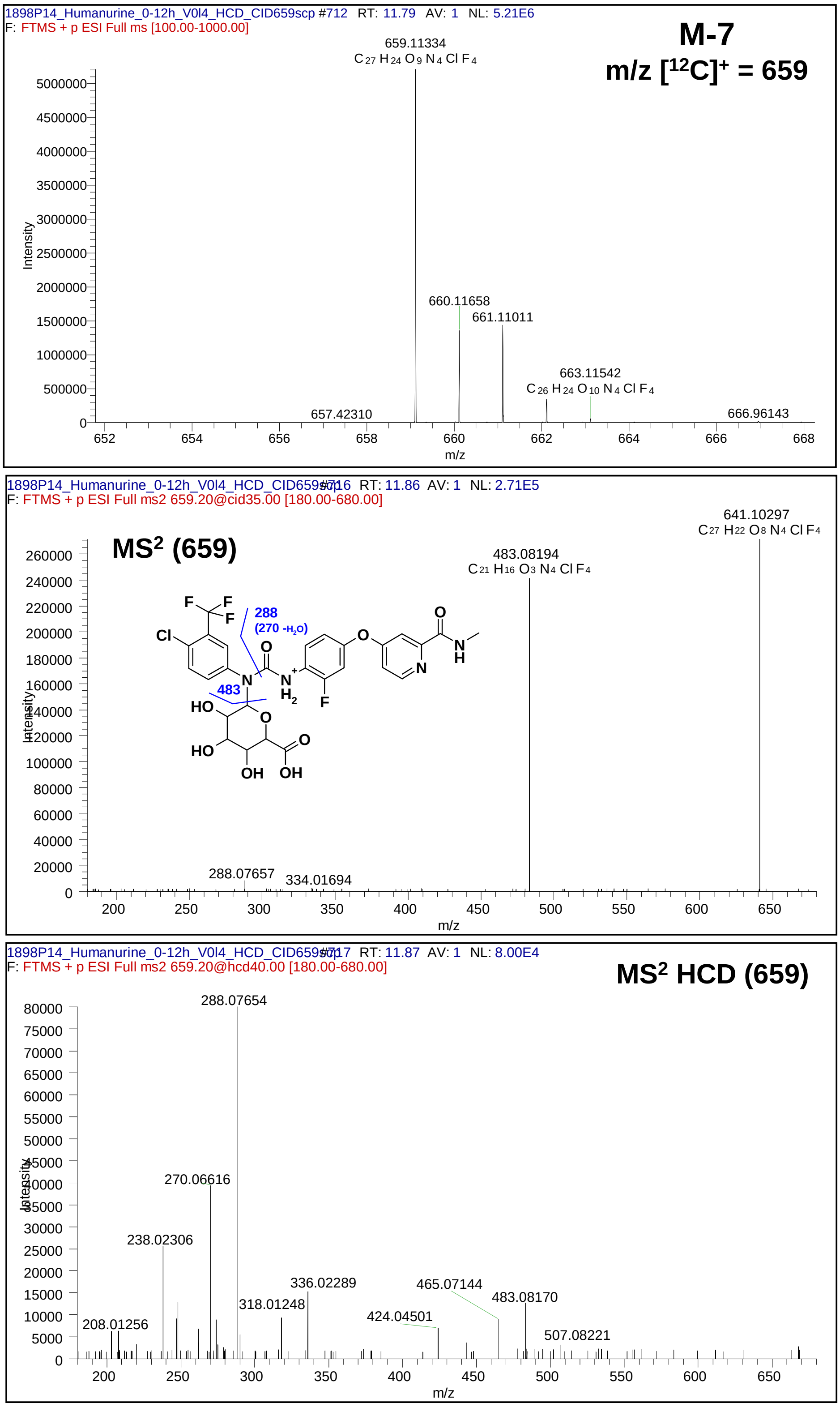

Fig. S9 Product ion spectrum of M-8

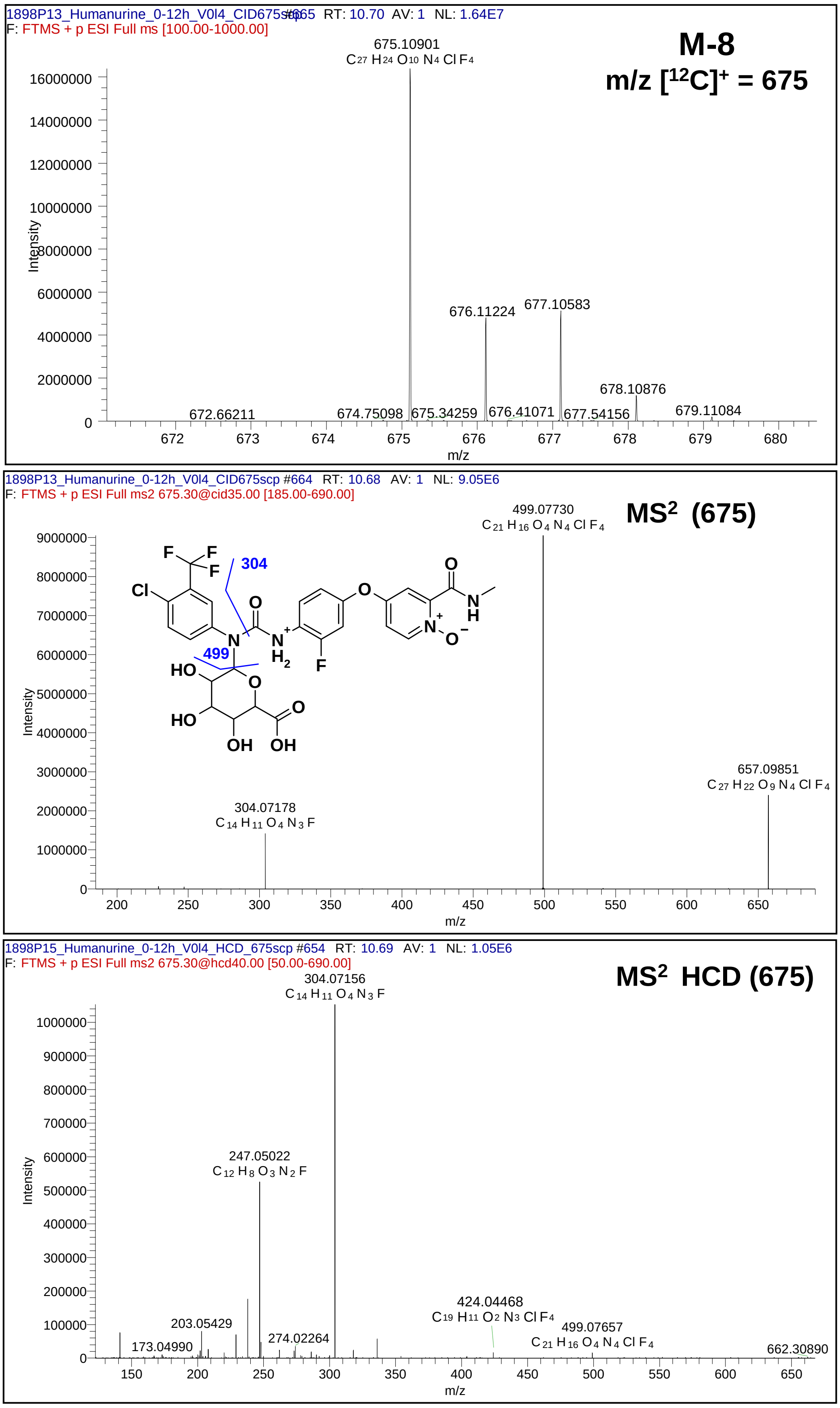

Fig. S10 NMR analysis of M-7

**Parent compound**	:	BAY 73-4506
**Metabolite**	:	M-7
**Sample**	:	GCM 2082-01
**Origin**	:	Human urine
**Chemical structure**	:	GCM2082-01 (M7) DMSO

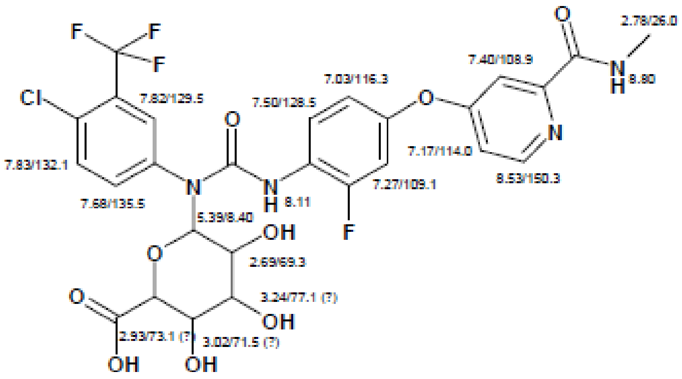

Chemical structure of **GCM 2082-01, M-7**

NMR, results:

^{1}H-NMR (DMSO-d_6, 600.13MHz):δ=8.80(q, J=4.9 Hz, 1H), 8.53(d, J=5.5Hz, 1H), 8.11(bs, 1H), 7.83(m, 2H), 7.68(d, J=8.9 Hz, 1H), 7.50(m, 1H), 7.40(d, J=2.4 Hz, 1H), 7.27(dd, J=10.8, 2.7 Hz, 1H), 7.17(dd, J=5.5, 2.3 Hz, 1H), 7.03(dd, J=8.7, 2.5 Hz, 1H), 2.78(d, J=4.9 Hz, 3H)

^{1}H,^{13}C-HSQC (DMSO-d_6, 600.13MHz,150.91MHz): δ=(8.53, 150.3), (7.83, 132.1; 7.82, 129.5), (7.68, 135.5), (7.50, 128.5), (7.40, 108.9), (7.27, 109.1), (7.17, 114.0), (7.03, 116.3), (2.78, 26.0)

Glucuronide part:
(5.39, 8.40), (3.24, 77.1), (3.02, 71.5), (2.93, 73.1), (2.69, 69.3)
